# Uric Acid Is Independently Associated with Diabetic Kidney Disease: A Cross-Sectional Study in a Chinese Population

**DOI:** 10.1371/journal.pone.0129797

**Published:** 2015-06-01

**Authors:** Dandan Yan, Yinfang Tu, Feng Jiang, Jie Wang, Rong Zhang, Xue Sun, Tao Wang, Shiyun Wang, Yuqian Bao, Cheng Hu, Weiping Jia

**Affiliations:** 1 Shanghai Diabetes Institute, Shanghai Key Laboratory of Diabetes Mellitus, Shanghai Clinical Center for Diabetes, Shanghai Jiao Tong University Affiliated Sixth People’s Hospital, Shanghai, China; 2 Shanghai Jiao Tong University Affiliated Sixth People’s Hospital, South Branch, Shanghai, China; University of Hong Kong, CHINA

## Abstract

**Background:**

Association between hyperuricaemia and chronic kidney disease has been studied widely, but the influence of uric acid on the kidneys remains controversial. We aimed to summarize the association between uric acid and diabetic kidney disease (DKD), and to evaluate the role of uric acid in DKD.

**Methods:**

We enrolled 3,212 type 2 diabetic patients in a cross-sectional study. The patients’ basic characteristics (sex, age, BMI, duration of disease, and blood pressure) and chemical parameters (triglycerides, total cholesterol, low-density lipoprotein cholesterol (LDL-c), high-density lipoprotein cholesterol (HDL-c), microalbuminuria, creatinine, and uric acid) were recorded, and the association between uric acid and DKD was evaluated.

**Results:**

In the 3,212 diabetic patients, the prevalence of diabetic kidney disease was higher in hyperuricaemic patients than in patients with normouricaemia (68.3% vs 41.5%). The prevalence of DKD increased with increasing uric acid (*p* <0.0001). Logistic analysis identified uric acid as an independent predictor of DKD (*p* <0.0001; adjusted OR (95%CI) = 1.005 (1.004–1.007), *p* <0.0001). Uric acid was positively correlated with albuminuria and creatinine levels (*p*<0.0001) but negatively correlated with eGFR (*p*<0.0001) after adjusting for confounding factors.

**Conclusions:**

Hyperuricaemia is a risk factor for DKD. Serum uric acid levels within the high-normal range are independently associated with DKD.

## Introduction

Serum uric acid is the end product of purine degradation in humans and great apes, in whom uricase expression disappeared during evolution. Uric acid can act as either an antioxidant [1] or an oxidant [2] depending on the chemical environment. According to previous epidemiological or experimental studies, serum uric acid is correlated with disorders such as obesity [3], diabetes mellitus [4], hypertension [5], cardiovascular disease [6] and chronic kidney disease [7,8], in which uric acid acts as an oxidant, inducing oxidative stress and endothelium dysfunction. Diabetic kidney disease (DKD) represents the leading cause of end-stage kidney disease in many countries [9] and has become a worldwide burden, prompting investigations into the factors, notably uric acid, related to the onset and progression of DKD [10]. Both animal [7] and human [11] studies have suggested that hyperuricaemia can induce hyalinosis and wall thickening of kidney preglomerular arterioles and can promote the progression of chronic kidney disease by regulating glomerular haemodynamics. However, not all observational studies [12,13] have described uric acid as a promoter in the context of chronic kidney disease. Thus, the influence of uric acid on the kidneys remains controversial, necessitating further investigation into the association between uric acid and DKD. In this study, we aimed to determine the incidence of DKD as well as the clinical parameters of kidney function to further evaluate the association between uric acid and DKD.

## Materials and Methods

### Ethics statement

This study was approved by the institutional review board of Shanghai Jiao Tong University Affiliated Sixth People’s Hospital in accordance with the principles of the second revision of the Declaration of Helsinki. Written informed consent was obtained from each patient.

### Participants

A total of 3,212 patients definitively diagnosed with type 2 diabetes were recruited from the Shanghai Diabetes Institute Inpatient Database. All subjects resided in Shanghai or nearby regions. Patients with cancer, hepatic disease or other coexisting illnesses including autoimmune kidney diseases, renal artery stenosis were excluded, and patients using medicines such as uric acid lowering agents, diuretics, salicylate, ethambutol, nicotinic acid, cyclosporin, or fructose that might influence the level of uric acid were excluded. Type 2 diabetes patients were diagnosed according to the 1999 criteria of the World Health Organization (WHO). Type 1 diabetes and mitochondrial diabetes were excluded by clinical, immunological and genetic criteria.

### Clinical measurements

Anthropometric and biochemical traits related to diabetes were extensively evaluated for all participants. Height (m) and weight (kg) were measured, and BMI was calculated as weight/height^2^. Blood pressure (mmHg) was measured in the right arm with the individual in seated position, using a mercury sphygmomanometer by the experienced medical staff, the measurements were repeated three times with five-minute intervals between them, and the averages of these measurements were used for further analysis. Serum uric acid, creatinine, triglyceride, total cholesterol, low-density lipoprotein cholesterol (LDL-c), high-density lipoprotein cholesterol (HDL-c) and creatinine levels were measured using a type 7600–020 Automated Analyser (Hitachi, Tokyo, Japan). Plasma glucose concentrations were measured by the glucose oxidase-peroxidase method using commercial kits (Shanghai Biological Products Institution, Shanghai, China). HbA1c values were determined by high performance liquid chromatography using a Bio-Rad Variant II haemoglobin testing system (Bio-Rad Laboratories, Hercules, CA, USA). Hyperuricaemia was defined as serum uric acid over 7mg/dl for males and serum uric acid over 6mg/dl for females [14].

#### DKD examination and diagnosis

Albuminuria was measured by scatter turbidimetry using the BN II System (Siemens Healthcare Diagnostics Products GmbH, Marburg, Germany). The estimated glomerular filtration rate (eGFR) was calculated using the modification of diet in renal disease study equation (MDRD) for the Chinese population [15]. Patients with albuminuria ≥ 30 mg/24 h or eGFR < 90 mL/min per 1.73 m^2^ were diagnosed with DKD.

### Statistical analysis

All analyses were performed using SAS9.2. Normality testing was performed, and variables with skewed distributions (BMI, duration, systolic blood pressure, diastolic blood pressure, triglyceride, total cholesterol, HDL-c, LDL-c, uric acid, and HbA1c) were analysed after logarithmic transformation. The data were summarised as the median (interquartile range) or mean±SD for continuous variables and as proportions for categorical variables. Differences in the clinical characteristics of the patients were assessed using the t-test or Kruskal Wallis test for continuous variables and the χ2 test for categorical variables. Multivariable logistic regression analysis was used to identify factors associated with DKD. Regression analysis was performed to evaluate the associations between uric acid and indexes of DKD. *P*-values <0.05 were considered significant.

## Results

Among the 3,212 total participants, 18.7% exhibited hyperuricaemia. We summarised the clinical characteristics of the participants according to their serum uric acid concentration in Table 1. BMI, systolic blood pressure, microalbuminuria, creatinine, and HbA1c were elevated relative to the normouricaemic group (*p*<0.0001), whereas eGFR was reduced (*p*<0.0001). These findings remained significant after classification by gender. As shown in Table 2, the prevalence of DKD correlated with elevated serum uric acid levels (*p*<0.0001). Furthermore, elevated serum uric acid levels were correlated with increased BMI, systolic blood pressure, diastolic blood pressure, microalbuminuria, creatinine, and triglycerides (*p*<0.0001). LDL-c and HDL-c were also elevated in patients with hyperuricaemia, but eGFR and HbA1c were decreased (*p*<0.0001).

**Table 1:** Clinical characteristics of the study subjects.

	Total			Male			Female		
	Normouricaemia	Hyperuricaemia	p value	Normouricaemia	Hyperuricaemia	p value	Normouricaemia	Hyperuricaemia	p value
n	2452	565	_	1310	253	_	1142	312	_
Age (y)	60.5±12.0	63.2±13.8	<0.0001	58.8±12.6	58.6±15.3	0.7312	62.9±11.0	67.0±11.2	<0.0001
BMI (kg/m^2^)	23.9(21.8,26.2)	25.5(23,27.7)	<0.0001	24(22,26.2)	25.5(23.1,27.7)	<0.0001	23.7(21.5,26.2)	25.5(22.8,27.7)	<0.0001
SP (mmHg)	130(120,145)	140(125,150)	<0.0001	130(120,140)	132.5(120,150)	0.0117	135(120,150)	140(130,157.5)	<0.0001
DP (mmHg)	80(75,90)	80(77,90)	0.0100	81(75,90)	80(80,90)	0.0351	80(70,85)	80(75,90)	0.0797
Uric acid (mg/dL)	4.8(4.0,5.6)	7.3(6.7,8.0)	<0.0001	5.2(4.4,6.0)	7.7(7.3,8.5)	<0.0001	4.4(3.7,5.0)	6.8(6.3,7.5)	<0.0001
Microalbumin (mg/24 h)	12.4(7.0,32.8)	22.1(8.5,87.6)	<0.0001	13.4(7.2,35.7)	24.6(8.5,104.7)	<0.0001	11.8(6.9,28.0)	19.7(8.2,73.1)	<0.0001
MDRD (ml/min 1.73 m^2^)	108.4(89.1,130.4)	84.0(57.7,106.8)	<0.0001	103.6(86.1,124)	80.8(55.9,104.6)	<0.0001	114.4(95.5,139.4)	87(58.3,107.8)	<0.0001
Creatinine (µmol/L)	65(55,78)	78(63,105)	<0.0001	73(64,84)	89(75,119)	<0.0001	56(48,64)	68.5(58,93)	<0.0001
LDL (mmol/L)	2.5(1.5,3.3)	2.6(1.8,3.4)	<0.0001	2.4(1.5,3.2)	2.5(1.6,3.2)	0.0486	2.6(1.5,3.4)	2.7(1.9,3.4)	0.0006
HDL (mmol/L)	1.5(1.1,4.3)	1.4(1.0,4.4)	0.1934	1.4(1.0,4.2)	1.4(0.9,4.2)	0.6428	1.6(1.2,4.5)	1.4(1.0,4.6)	0.0708
TC (mmol/L)	3.7(1.2,5.0)	3.8(1.1,5.0)	0.4708	3.5(1.1,4.8)	3.5(1.1,4.8)	0.5047	3.9(1.3,5.2)	4.0(1.2,5.2)	0.4554
Triglyceride (mmol/L)	2.1(1.3,3.0)	2.3(1.6,3.3)	<0.0001	2.1(1.3,2.9)	2.3(1.5,3.3)	0.0002	2.1(1.3,3.0)	2.5(1.7,3.3)	<0.0001
FPG (mmol/L)	9.7 (7.4,12.4)	9.0 (7.1,11.6)	0.0014	9.7 (7.4,12.4)	8.8 (6.8,11.9)	0.0068	9.8 (7.3,12.4)	9.1 (7.3,11.5)	0.0703
2h PG (mmol/L)	14.5 (11.2,17.8)	13.3 (10.4,16.4)	0.0002	14.6 (11.2, 17.8)	13.1 (10.4,16.7)	0.0063	14.5 (11.2,17.9)	13.5 (10.6,16.3)	0.0079
HbA1c (%)	9.1(7.5,10.8)	8.1(6.8,10.0)	<0.0001	9.3(7.6,11.0)	8.2(6.6,10.6)	<0.0001	8.9(7.3,10.4)	8.0(6.9,9.7)	<0.0001
HbA1c (mmol/mol)	76(58,95)	65(51,86)	<0.0001	78(60,97)	66(49.7,92)	<0.0001	74(56,90)	75(52,83)	<0.0001

Data are shown as mean±SD or the median (interquartile range). BMI: Body mass index. SP: Systolic blood pressure. DP: Diastolic blood pressure. MDRD: Modification of diet in renal disease. LDL: Low-density lipoprotein cholesterol. HDL: High-density lipoprotein cholesterol. TC: Total cholesterol. FPG: fasting plasma glucose. 2h PG: 2 hour postprandial glucose.

**Table 2:** Index trends according to the uric acid category.

	Level 1(0–3mg/dl)	Level 2(3–4mg/dl)	Level 3(4–5mg/dl)	Level 4(5–6mg/dl)	Level 5(6–7mg/dl)	Level 6(≥7mg/dl)	p for trend
n	148	474	806	708	509	372	_
SP (mmHg)	130(120,140)	130(120,140)	130(120,145)	130(120,146)	138(120,150)	140(120,150)	<0.0001
DP (mmHg)	80(70,80)	80(70,85)	80(75,90)	80(75,90)	80(80,90)	80(80,90)	<0.0001
Uric acid (mg/dl)	2.6(2.2,2.8)	3.6(3.4,3.8)	4.6(4.3,4.8)	5.5(5.2,5.7)	6.4(6.2,6.7)	7.7(7.3,8.5)	<0.0001
Microalbumin (mg/24 h)	12.9(8.1,30.8)	12.0(6.6,26.2)	11.1(6.7,27.1)	12.4(7.1,38.3)	16.1(7.6,50.6)	26.4(9.5,126.4)	<0.0001
MDRD (ml/min 1.73 m^2^)	133.4(104.4,165.5)	119.1(100.7,143.1)	111.2(93.4,134.9)	101.6(84.5,118.9)	94.2(74.8,114.8)	77.8(49.8,103.0)	<0.0001
Creatinine (μmol/L)	50(44,64)	58(50,68)	61(53,73)	70(60,82)	74(63,89)	87(72,120)	<0.0001
LDL (mmol/L)	3.2(2.0,3.7)	2.5(1.4,3.3)	2.4(1.5,3.3)	2.5(1.5,3.4)	2.6(1.8,3.3)	2.6(1.8,3.3)	0.0003
HDL (mmol/L)	1.7(1.2,4.2)	1.6(1.2,4.0)	1.5(1.1,4.3)	1.6(1.1,4.5)	1.3(1.0,4.4)	1.4(1.0,4.3)	0.0038
TC (mmol/L)	3.6(1.4,5.0)	3.9(1.4,5.0)	3.7(1.2,4.9)	3.6(1.1,5.1)	3.9(1.1,5.0)	3.7(1.1,4.9)	0.1505
Triglyceride (mmol/L)	1.7(0.9,2.6)	1.8(1.0,2.7)	2.1(1.2,2.9)	2.3(1.5,3.1)	2.3(1.6,3.2)	2.3(1.6,3.3)	<0.0001
HbA1c (%)	10.0(8.4,11.7)	9.5(7.7,11.3)	9.3(7.6,10.8)	8.8(7.3,10.4)	8.2(6.9,10.0)	8.2(6.8,10.3)	<0.0001
HbA1c (mmol/mol)	86(68,104)	80(61,100)	78(60,95)	73(56,90)	66(52,86)	66(51,89)	<0.0001

Data are shown as the median (interquartile range). SP: Systolic blood pressure. DP: Diastolic blood pressure. MDRD: Modification of diet in renal disease. LDL: Low-density lipoprotein cholesterol. HDL: High-density lipoprotein cholesterol. TC: Total cholesterol.

**Fig 1:**
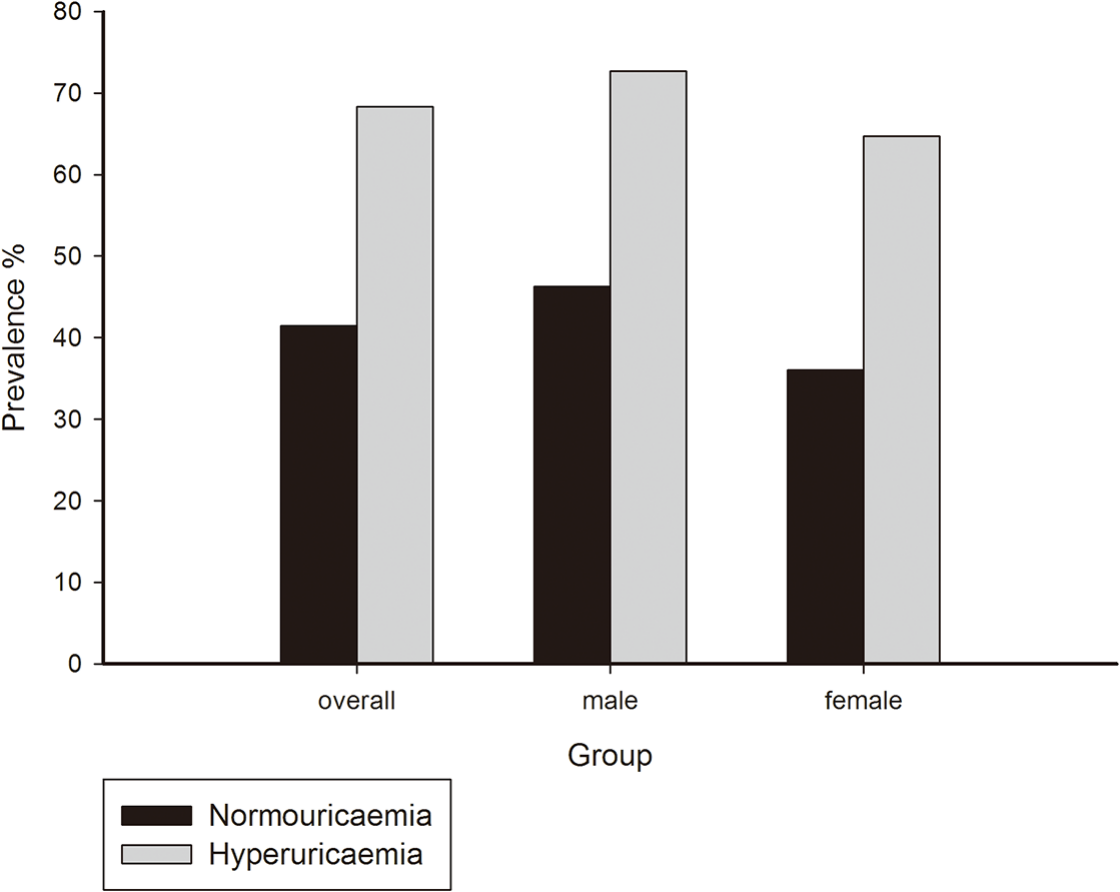
Prevalence of DKD in different groups. The prevalence of DKD in hyperuricaemia was significantly higher than normouricaemia, which was calculated in the whole group, male, female participants separately. *p<0.05 by χ2 test.

The overall prevalence of DKD was significantly higher (68.3% vs 41.5%, *p*<0.0001) in the hyperuricaemic group than in the normouricaemic group, and the difference remained significant after classification by gender (Fig 1). Logistic regression analysis revealed that serum uric acid, whether treated as a continuous (OR = 1.381, 95%CI = 1.293–1.476, *p* <0.0001) or a stratified variable (OR = 1.435, 95%CI = 1.335–1.543, *p* <0.0001), remained strongly associated with DKD after adjusting for confounding factors including sex, age, BMI, duration of diabetes, blood pressure, HbA1c and serum lipids. We also investigated the association of serum uric acid with DKD defined by reduced eGFR alone or albuminuria alone by logistic regression analysis, and serum uric acid was associated with DKD significantly in both conditions (OR = 1.009, 95%CI = 1.007–1.010, *p*<0.0001; OR = 1.003, 95%CI = 1.002–1.004, *p*<0.0001) after adjusting for sex, age, BMI, duration of diabetes, blood pressure, HbA1c and serum lipids.

**Fig 2:**
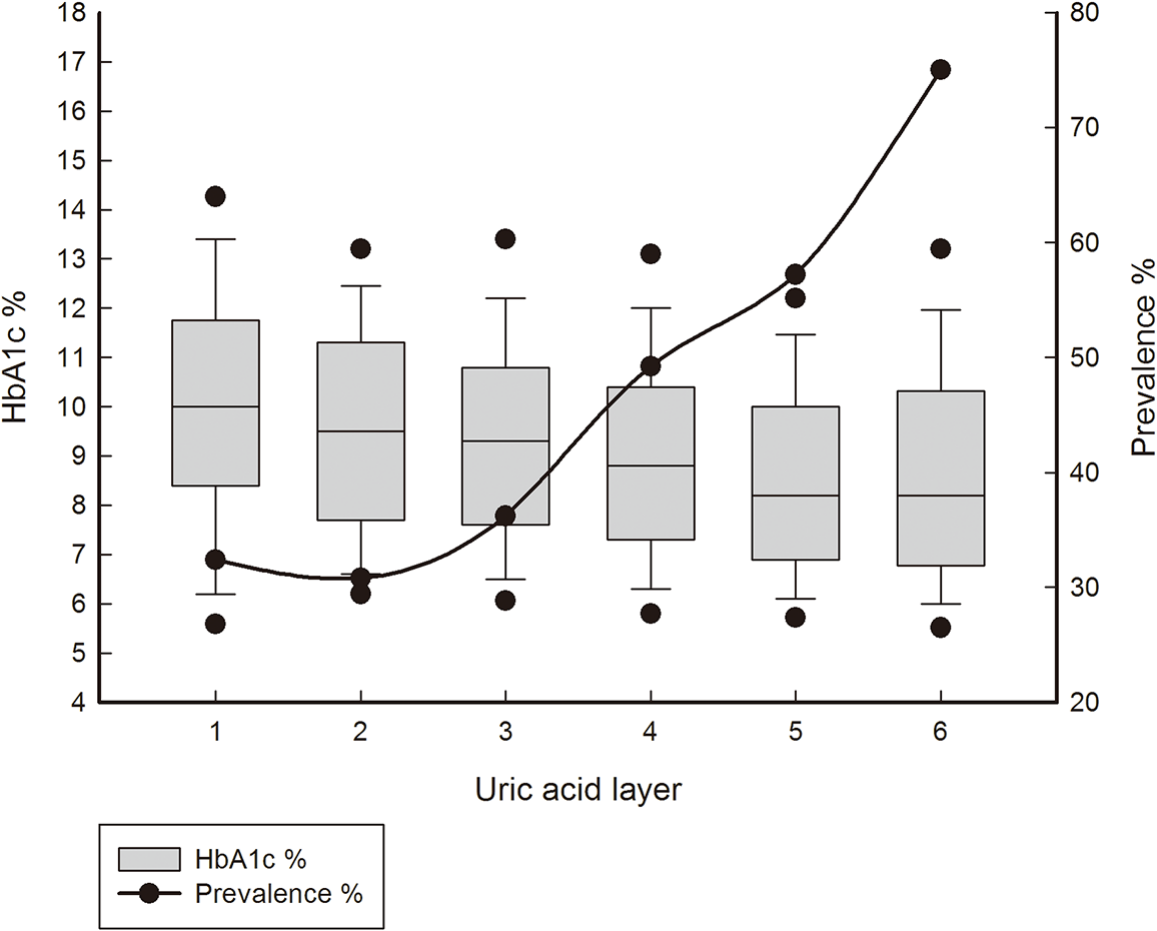
Association between uric acid and HbA1c levels and the prevalence of DKD. The uric acid was categorized into 6 levels and the HbA1c levels, the prevalence of DKD were calculated separately. HbA1c levels were shown by box plots, and the prevalence of DKD was shown by simple straight line & scatter. Increased serum uric acid was correlated with decreased HbA1c and an elevated prevalence of DKD (*p*<0.0001).

As DKD is a syndrome characterised by the presence of urine albumin excretion, glomerular lesions, loss of GFR and increased serum creatinine, and because HbA1c is related to the progression of DKD [16], we further evaluated the association between the above variables and uric acid to confirm the role of uric acid. After adjusting for confounding factors, including sex, age, BMI, duration of diabetes, blood pressure, HbA1c and serum lipids, the microalbuminuria and creatinine levels were significantly correlated with serum uric acid (β±SE = 0.64±0.11for log transformed microalbumin and uric acid, *p*<0.0001; β±SE = 0.33±0.02 for log transformed creatinine and uric acid, *p*<0.0001), whereas the eGFR level was inversely correlated with serum uric acid (β±SE = -0.4±0.03 for log transformed eGFR, *p*<0.0001). As shown in Fig 2, increased serum uric acid was correlated with decreased HbA1c and an elevated prevalence of DKD (*p*<0.0001).

## Discussion

According to our current results, the prevalence of DKD was significantly elevated in hyperuricaemic participants and increased with increasing uric acid levels. The increase in the prevalence of DKD was more obvious in male patients than in females. Serum uric acid was independently associated with DKD in logistic regression analysis after adjusting for confounding factors, including age, sex, BMI, duration of diabetes, blood pressure, serum lipids, and HbA1c. Multiple linear regression revealed that serum uric acid was positively correlated with microalbuminuria and creatinine levels but negatively correlated with eGFR. Furthermore, increased levels of uric acid were correlated with decreased HbA1c and a significantly elevated prevalence of DKD.

Historically, uric acid was recognised as a consequence of renal insufficiency, but recent studies have suggested that the causal relationship may have been inverted [17]. Although some previous studies [12,13] revealed that hyperuricaemia is not associated with chronic kidney disease, numerous observational studies [3,18–21] indicate that uric acid is independently associated with chronic kidney disease. According to a cross-sectional study involving 2,108 Chinese patients with type 2 diabetes [22], high-normal uric acid is positively correlated with albuminuria and impaired renal function. During a 5-year follow-up of 1,449 type 2 diabetic patients with normal kidney function, hyperuricaemia was identified as an independent risk factor for the development of DKD [19]. Our results are consistent with the above studies, although these studies were performed in various areas and employed different designs and sample sizes.

As previous studies have revealed, there are several mechanisms underlying the association between uric acid and DKD. Within the serum, uric acid can react with various oxidants and act as an antioxidant [23]. When serum uric acid is transported into vascular smooth muscle cells, it can cause impaired NO production and release [24], inducing endothelial dysfunction and promoting the progression of DKD [25]. By activating the intracellular MAPK pathway and nuclear transcription factors (NF-κB and AP-1), uric acid can cause the proliferation of vascular smooth muscle cells [26]. Furthermore, uric acid can induce oxidative stress [27,28], thus causing preglomerular arteriolar damage. This damage can change glomerular haemodynamics and results in chronic renal damage. A clinical biopsy-based study [11] revealed that serum uric acid may cause the progression of chronic kidney disease via renal arteriolopathies such as hyalinosis and wall thickening. In animals fed with oxonic acid, an uricase inhibitor, elevated uric acid levels promoted chronic kidney disease via endothelial dysfunction, local activation of the renin–angiotensin system, oxidative stress, and preglomerular arteriolar damage [7]. Both clinical [29,30] and animal studies [7] have indicated that the use of allopurinol, a xanthine oxidase inhibitor, can lower uric acid levels and slow the progression of chronic kidney disease.

Given that hyperuricaemia is a risk factor for type 2 diabetes [4], the inverse correlation between uric acid and HbA1c seems counterintuitive, but a previous study [31] also obtained the same result. According to previous studies, the transporters of glucose [32] and uric acid [33] in the kidney may underlie this result. For example, GLUT9, a facilitative glucose transporter that is expressed at the apical membrane of kidney tubular cells, transports both uric acid and D-glucose, as demonstrated by transporter inhibition experiments in cultured cells [34] and other studies [35]. Increased glycosuria can lower serum uric acid by decreasing uric acid reabsorption [34]. Therefore, a decrease in blood glucose could reduce glycosuria, thus increasing the reabsorption of uric acid and inducing hyperuricaemia.

The limitations of this study require further comment. First, as our study is localised in Shanghai and nearby regions and the sample size is small, our findings may be specific to Chinese patient and may exhibit inherent bias. Second, our conclusion that uric acid plays a role in DKD rests on the combination of our results with those of a previous, due to limitations imposed by the cross-sectional nature of this study, we are unable to confirm a causal relationship between uric acid and DKD. Thus, further prospective, experimental and/or interventional studies are needed. Third, as a complex disease, many factors may influence the outcome of DKD, we could not exclude all the confounding factors in our study, further studies are needed to explore the causal relationship between uric acid and DKD.

In conclusion, serum uric acid is independently associated with DKD and acts as an indicator of DKD.
